# Entrepreneurship Resilience: Can Psychological Traits of Entrepreneurial Intention Support Overcoming Entrepreneurial Failure?

**DOI:** 10.3389/fpsyg.2021.707803

**Published:** 2021-09-14

**Authors:** Hong Zhao, Ardy Wibowo

**Affiliations:** ^1^International College of Cultural Education, Northeast Agricultural University, Harbin, China; ^2^Department of Information Management, College of Management, National Kaohsiung University of Science and Technology, Kaohsiung, Taiwan; ^3^Faculty of Economics and Business, Alma Ata University, Yogyakarta, Indonesia

**Keywords:** psychological traits, learning from failure, recovery capabilities, entrepreneurial engagement, opportunity recognition, entrepreneur failure

## Abstract

Entrepreneurial failure (EF) can occur due to aspects beyond the control of an entrepreneur, even if planning and calculations have been thorough. This research proposes a framework to illustrate how entrepreneurs cope with failure, based on the psychological characteristics that lead them to become entrepreneurs. Entrepreneurial self-efficacy and internal locus of controls measure the perceived learning from failure and recovery ability that can support continued entrepreneur engagement and new opportunity recognition after a failure. This study applied Partial Least Square to calculate and evaluate data from 146 respondents to an online questionnaire survey. The analysis shows that the psychological characteristics represented by entrepreneurial self-efficacy and internal locus of control can influence the willingness of entrepreneurs to learn from failure and increase their ability to recover. This can increase the willingness to continue in entrepreneurship and help them to recognize new opportunities. However, recovery ability does not support entrepreneurial self-efficacy or new opportunity recognition because the ability to recover may vary among the entrepreneurs, depending on many factors.

## Introduction

Entrepreneurial failure (EF) is very likely to occur due to factors beyond the control of an entrepreneur, despite planning and calculations beforehand (Ucbasaran et al., 2013; Yamakawa and Cardon, 2015; Khelil, 2016). Therefore, the ability of an entrepreneur to come back from failure should be better understood. This behavior represents the ability of an entrepreneur to learn from failures. It also illustrates the resilience of a person to remain an entrepreneur and continue in business development, despite a failure.

Entrepreneurial failure has emerged as an important research topic (Jenkins and McKelvie, 2016). In general, researchers define EF as under-expected business performance that drives the entrepreneurs into the failure condition psychologically. Cope (2011) mentions that EF is a continuum that spans from causes to consequences. Previous research has discussed how an entrepreneur should behave after failure, providing a comprehensive and critical study of EF concepts (Ucbasaran et al., 2013) and theoretical viewpoints (Dias and Teixeira, 2017). Researchers have also found the relation of EF on young entrepreneurs (Khelil, 2016), specifically on Small-Medium Enterprises (SMEs) (Michael and Combs, 2008), and start-up businesses (Artinger and Powell, 2016). However, there has been a less qualitative examination of entrepreneurial behavior after a failure and entrepreneurial resilience after failure.

Internal individual factors can influence the EF (Yamakawa and Cardon, 2015; Walsh and Cunningham, 2017), and they can be attributed to the entrepreneur on a personal basis. In contrast, previous research has widely explored factors underlying the intention of a person to be an entrepreneur, especially psychological factors. Strong empirical results emphasize that entrepreneurial self-efficacy and internal locus of control play a critical role as a personal basis underlying entrepreneurial intention (Shinnar et al., 2014; Hsiao et al., 2016; Tsai et al., 2016; Farrukh et al., 2017; Asante and Affum-Osei, 2019). These traits are inherent in behavior, beliefs, and cognitive models of entrepreneurs as internal personalities. Moreover, Felin and Foss (2006) pointed out that EF may be influenced by internal factors of individual-level variables. Thus, this research considers entrepreneurial self-efficacy and internal locus of control as psychological traits that need to be proven, as both a foundation when people start as entrepreneurs and as a foundation for resilience after failure.

The impact and transition of failure include steps taken by entrepreneurs after a failure (Omorede, 2020) as they look for ways to recover by understanding what factors led to that failure. The mechanism of recovery from failure is highly influenced by feelings and emotional reactions (Cope, 2011). Furthermore, entrepreneurs learn from their previous failures through sense-making and reflection. They also may consider the importance of not making the same mistakes or making the same choices that led to their previous failure before embarking on new ventures (Omorede, 2020). Thus, the constructs of recovery capabilities and perceived learning from failure are considered a result of failure of an entrepreneur.

Actions taken after the entrepreneurs have accepted and come back from their failures are part of the outcomes of failure. Having learned from the failure, entrepreneurs can go on to consider other business opportunities and exploit new opportunities using their existing knowledge and networks (Omorede, 2020). This may allow entrepreneurs to better take advantage of new business opportunities (Amankwah-Amoah et al., 2016). The process of launching a new venture plan is a means for an entrepreneur to recover from past failures (Amankwah-Amoah et al., 2016; Walsh and Cunningham, 2017). Moreover, launching a new venture or enterprise shows they want to continue as an entrepreneur by leveraging their experience.

This study integrates the construct to produce useful implications for parties or stakeholders related to entrepreneurs and to address the limitations of previous research. Yu et al. (2014) stated that some individual characteristics influence learning outcomes. At the organizational level of analysis, those characteristics and the learning outcomes can influence future behavior, entrepreneurial choices, and results. This study considers personality traits that represent the internal locus of control and entrepreneurial self-efficacy of an entrepreneur as the traits that underlie the entrepreneurs when they faced failure. It then evaluates how they will affect their capacity to derive learning from mistakes and their ability to recover. It also investigates their influence on failure outcomes, which can support new opportunity recognition and continuance of entrepreneurship engagements. Overall, this study provides insight into physiological traits that underlie the intention of an individual to be an entrepreneur and may also underlie the ability of an entrepreneur to rise from failure.

## Literature Review

### EF

Entrepreneurial failure is explained as a psychological and economic phenomenon arising due to underperformance of an organization or falling below the expectations of an entrepreneur, so the entrepreneur enters psychological conditions of failure (Khelil, 2016; Dias and Teixeira, 2017). EF refers to the process of entrepreneurial failure from the perspective of entrepreneurs, because they are the main ones affected by the failure (Klimas et al., 2020). EF is an important aspect of the entrepreneurial process (McGrath, 1999; Zahra and Dess, 2001), which is a personal endeavor (Stevenson and Jarillo, 1990), so the focus of EF is on the individual level. Otherwise, the term “exit” refers to the failure of a corporation (Jenkins and McKelvie, 2016). Also, EF may be caused by internal factors such as a lack of experience and making unrealistic decisions, as well as external factors such as financial constraints (Larson and Clute, 1979; Pretorius, 2008; Ucbasaran et al., 2013).

### Psychological Traits

The driving factors of the decision of an individual to start a business are personality traits (McClelland, 1961; Brockhaus, 1980; Krueger et al., 2000), and differences in personality traits between entrepreneurs and non-entrepreneurs are assumed to be important preconditions for entrepreneurship (Utsch and Rauch, 2000). According to Learned (1992), some people have a combination of psychological characteristics and background factors that make them more likely to attempt to start a company. Internal locus of control and entrepreneurial self-efficacy are psychological traits that underlie the intention of an individual to be an entrepreneur in a variety of contexts and settings (Hsiao et al., 2016; Tsai et al., 2016; Farrukh et al., 2017; Asante and Affum-Osei, 2019). Accordingly, these variables have been proven to be strong predictors for entrepreneur intention.

Moreover, entrepreneurial self-efficacy is a key indicator in addition to personality traits “because it refers to cognitive evaluations of personal capabilities in the specific task of entrepreneurship, both individual and contextual” (Chen et al., 1998; McGee et al., 2009). Furthermore, entrepreneurial efficacy has been recognized as a key driver for the intentions and success of entrepreneurs, particularly for start-up entrepreneurs (Naktiyok et al., 2009; Drnovšek et al., 2010; Shinnar et al., 2014; Tsai et al., 2016).

Meanwhile, regarding the internal locus of control, Rotter (1990) argued that the internal locus of control is the perception of an individual about an event that occurs, and it depends on the behavior or the characteristics of the individual. Internal locus of control has been proven to be a favorable predictor of entrepreneurial intention in numerous studies (Baldegger et al., 2017). Brunel et al. (2017) found that individuals with an internal locus of control feel they will succeed in entrepreneurship. Therefore, psychological characteristics, such as locus of control, play an important role in developing entrepreneurial intention.

Entrepreneurial self-efficacy and internal locus of control are considered important in personality theories of entrepreneurship. Thus, this research regards internal locus of control and entrepreneurial self-efficacy as strong factors that underlie the entrepreneurship intention of an individual, and it needs to be proven whether these factors can maintain the engagement of entrepreneurs to seek new opportunities.

#### Self-Efficacy

Self-efficacy was proposed by Bandura (1977) to describe the belief of a person in his/her ability to complete tasks. The development of intentions is thought to be preceded by self-efficacy. Individuals who believe that they have the potential to accomplish a task are more likely to develop the desire to accomplish it. In contrast, individuals who feel that they lack the potential to accomplish an objective are less likely to make plans to pursue that goal.

According to Scherer et al. (1989), people who believe that their parents are high achievers are more likely to believe that they should launch their own company than those who believe that their parents are low achievers or who have no such role models. Offspring of entrepreneurs often assume that they have a higher degree of competence when it comes to performing the tasks necessary to start a company.

Other studies argue that self-efficacy can be linked to entrepreneurship education as a predictor of entrepreneurial intention (Marques et al., 2012; Ndofirepi, 2020), behavioral approach (Ferreira et al., 2012), familial factors (Altinay et al., 2012; Farrukh et al., 2017), demographic characteristics (Nga and Shamuganathan, 2010; Shinnar et al., 2014), and as applied from the point of view of students (Dinis et al., 2013; Nasip et al., 2017).

#### Internal Locus of Control

Rotter (1954) used the viewpoint of locus of control to investigate personality traits. He considered that people who have an internal locus of control feel that their destiny is determined by their efforts and that they can control their fate. Conversely, people with an external control position believe that their fate is determined by chance or luck and is beyond their control (Lii and Wong, 2008). In addition, Luthans et al. (2006) indicated that people with an internal locus of influence are more likely to face difficulties and obstacles positively, finding meaningful solutions to overcome problems. Individuals with an internal locus of control are more motivated to succeed than those with an external locus of control, so when faced with a challenge, they are more motivated to learn and develop their skills and expertise.

Empirical studies on how locus of control affects entrepreneurial activities, particularly the motives to launch a business, have found conflicting results. For example, there have been studies on the entrepreneurial intentions of a small group of MBA students, showing that there is no difference between those with an entrepreneurial inclination and those without an entrepreneurial inclination (Chye Koh, 1996). Furthermore, some studies have found that there is no statistically significant relationship between entrepreneurial intention and locus of control (Gurel et al., 2010). Nevertheless, some previous studies have found that the inner locus of control is a driving factor for entrepreneurship (Ang and Hong, 2000).

### The Impact of Failure

When an entrepreneur fails, he/she incur a variety of outcomes, including social, financial, and physiological costs (Cope, 2011; Shepherd and Haynie, 2011; Shepherd et al., 2011). The perception of failure of an individual occurs when he/she thinks he/she has failed (Khelil, 2016; Dias and Teixeira, 2017), and this is an individual perception (Jeng and Hung, 2019). Through the psychological learning process, entrepreneurs can understand and learn from circumstances that bring about failure. Entrepreneurs engage in sense-making when they examine the failure to determine why and how their company failed. Therefore, this process could lead them to learn from failure by paying attention to the decisions, aspects, or competencies leading to EF (Omorede, 2020).

This study considers perceived learning from failures and recovery capability as the impact from failure. The recovery phase is the process of healing, which necessitates some psychological distance to overcome failure emotions (Klimas et al., 2020). As a result of an unsuccessful business plan, entrepreneurs can gain new knowledge, skills, and experience, and it is critical to recognize failure as a part of the learning process and understand it as a part of the mechanism that underpins the dynamic sense-making process (Shepherd et al., 2011). Singh et al. (2007) used multiple frameworks to analyze rich interview data and find evidence for grief recovery following EF. Furthermore, entrepreneurs can learn valuable lessons from their failures when they try to focus on and make sense of them to make adjustments and enhancements to their attitudes so as to prevent them from repeating the past errors (Cannon and Edmondson, 2001; Politis and Gabrielsson, 2009).

### Failure Outcomes

Entrepreneurs who learn from their mistakes and gain new experience can take advantage of new opportunities or launch a new business after their EF (Yamakawa and Cardon, 2015). Entrepreneurs who have learned from failures and gained new ideas can look for other market prospects with the skills and networks that they have developed (Amankwah-Amoah et al., 2016). Such entrepreneurs can progress to see new niches and areas for development (Atsan, 2016).

Many entrepreneurs who have experienced and learned from failures use this experience to start a new firm, and a better method of forming an organization helps ensure that they will use their past failures to recover (Amankwah-Amoah et al., 2016; Walsh and Cunningham, 2017). Entrepreneurs should learn to emerge from the haze of EF, recognize that the company has closed down, avoid stigma, and then recover. Table 1 provides the operational definition of each construct involved in this research.

**Table 1:** Operational definition.

**Construct**	**Definition**	**References**
Entrepreneurial self-efficacy	Confidence in one's ability to complete a task	Bandura, 1977
Internal locus of control	Individuals who have an internal locus of control think that the amount of effort they put in determines their success or failure and that they have control over their fate	Rotter, 1954
Perceived learning from failure	The cognitive capacity of entrepreneurs to gain new insights from prior experiences of failure to find and exploit new opportunities	Corbett, 2007
Recovery capabilities	The individual's ability to quickly process thoughts about the company's losses no longer elicits negative emotional reactions	Shepherd, 2003
Continuance of entrepreneurship engagement	Entrepreneurs' ability to move forward and seek other business opportunities by leveraging their existing knowledge and networks	Omorede, 2020
New opportunity recognition	Being aware of potential business ideas and gathering information on new product or service ideas	Kuckertz et al., 2017

## Development of Hypotheses

Emotional reactions and consequences are frequently associated with firm failure. The psychological and emotional experience following failure is a significant aspect of the experience (Omorede, 2020). When they fail, they experience anger, grief, disappointment, despair, sadness, regret, and other negative emotions. This research showed that the failed business exacted an “emotional toll” on the entrepreneurs. Nevertheless, after they had failed, hope, pride, and confidence were expressed by some entrepreneurs (Cope, 2011). For example, their loss may have provided them with a deeper perspective on their failure (Byrne and Shepherd, 2015). Therefore, this study assumes that the emotional state of an entrepreneur after experiencing failure is created or appears to be based on the psychological characteristics of each individual.

Omorede (2020) identified that the process of recovery from failure is primarily influenced by emotions and emotional reactions. Furthermore, they were able to learn from their previous experiences because of the emotional cost. They also emphasized the importance of not making the same mistakes or similar decisions that led to the failure of their previous business when starting new ventures. In this study, entrepreneurial self-efficacy and internal locus of control are the psychological characteristics of every entrepreneur that initially support their intention to become entrepreneurs. Thus, this research proposes H1–H4 as follows:

H1: Entrepreneurial self-efficacy has a positive impact on the perceived learning from failure.

H2: Entrepreneurial self-efficacy has a positive impact on recovery capability.

H3: Internal locus of control has a positive impact on perceived learning from failure.

H4: Internal locus of control has a positive impact on recovery capability.

Entrepreneurs claim that they learned from their past experiences through reflection and sense-making through the recovery process (Omorede, 2020). They learned more about their business and the reasons for their failure, as well as their external relationships and networks, and how to run a business more effectively in the future. Thus, H5 is proposed as follows:

H5: Recovery capability has a positive impact on perceived learning from failure.

Many entrepreneurs who suffer and learn from failures use their unsuccessful experiences to start up a new firm, and these findings suggest that one way for entrepreneurs to recover from previous failures is to start a new business (Amankwah-Amoah et al., 2016; Walsh and Cunningham, 2017). Entrepreneurs need to separate themselves from the emotion of EF, accept the fact that the company has failed, and avoid stigma, in order to successfully launch a new business (Walsh and Cunningham, 2017). Furthermore, entrepreneurs believe that their past failures have helped them to direct their potential business and career paths, as well as the decisions that led to them (Dias and Teixeira, 2017). Thus, this research proposes H6 and H7 as follows:

H6: Perceived learning from failure has a positive impact on the continuance of entrepreneurship engagement.

H7: Recovery capability has a positive impact on the continuance of entrepreneurship engagement.

After gaining new experiences and learning from failure, many entrepreneurs go on to pursue other business opportunities by exploiting their existing expertise and networks. This enabled the entrepreneurs to capitalize on new business opportunities (Amankwah-Amoah et al., 2016). Furthermore, venture capitalists are interested in investing in entrepreneurs who have been unsuccessful because they believe that the ability of entrepreneurs to find new business opportunities is more important than the past failure (Cope et al., 2004). In other words, venture capitalists seek entrepreneurs who made a full recovery from a failure and find new opportunities that leverage their experience. Thus, this research proposes H8 and H9 as follows:

H8: Perceived learning from failure has a positive impact on new opportunity recognition.

H9: Recovery capability has a positive impact on new opportunity recognition.

## Research Method

This study used a questionnaire to collect responses from former unsuccessful failed entrepreneurs. This research limits itself to the individual level of analysis, which means that the event of failure is experienced only by the entrepreneur, not as an organization or a company. The initial questions were asked to make sure that all the participants meet our criteria, “Have you experienced failure in entrepreneurship?” and “what kind of costs of business failure affect you? The financial cost, social cost, and/or psychological cost?” If the participants answered “No” in the first question, the assessment form will stop since these participants did not meet our criteria. The second question was developed from the findings of Ucbasaran et al. (2013), which state that the three types of aftermath to EF are financial, social, and psychological. This questionnaire aims to make the participant recall that event of failure and to ensure that they understand what is the “failure” that we mean.

**Figure 1:**
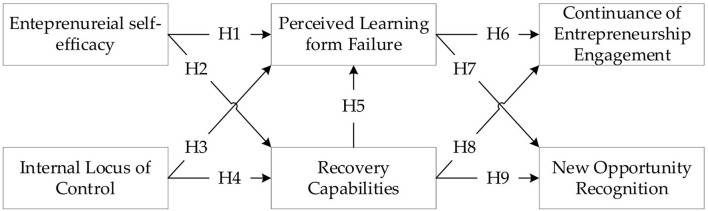
Research framework.

The questionnaire had two sections for demographic information and measurement questions. The overall framework is based on the model previously studied, as shown in Figure 1, and the items in the questionnaire are based on verified past research scales. The questionnaire was verified by several researchers with extensive experience in entrepreneurism. The validity of the contents of the questionnaire was then double-checked by them. The seven-point Likert scales were used to improve the accuracy of the scales (Churchill and Peter, 1984). Table 2 shows the measurement items adopted in this study.

**Table 2:** Measurement items.

**Item**	**Questions**
**Entrepreneurial self-efficacy (McGee et al.**, **2009****)**	
ESE1	I am able and confident in brainstorming (coming up with new ideas for a product or service)
ESE2	I can make a plan and estimate customer demand for a new product or service
ESE3	I can clearly and concisely explain verbally or in writing my business idea in everyday terms
ESE4	I can deal with and solve effectively day-to-day problems and crises
ESE5	I can manage the financial assets of my business
**Internal locus of control (Mueller and Thomas**, **2001****; Indarti and Krinstiansen**, **2003****)**	
ILC1	My life is determined by my actions
ILC2	When I get what I want, it is usually because I worked hard for it
ILC3	Whether or not I am successful in life depends mostly on my ability
ILC4	I feel in control of my life
ILC5	Diligence and hard work usually lead to success
**Perceived learning from failure (Shepherd et al.**, **2011****; Boso et al.**, **2019****; Liu et al.**, **2019****)**	
PLF1	I am applying what I learned from my previous failure experience in my new business
PLF2	I realize the mistakes that we made that led to the failure of our last venture
PLF3	I have learned to better manage the new venture since the last failed venture
PLF4	I am now alert to the performance feedback
PLF5	I have learned to better execute a business's strategy
**Recovery capabilities (Argentzell et al.**, **2017****)**	
REC1	My experiences have changed me for the better
REC2	I have been able to come to terms with things that have happened to me in the past and move on with my life
REC3	I am strongly motivated to get better
REC4	I can recognize the positive things I have done
REC5	I can make sense of my distressing experiences (dropped)
**Continuance of entrepreneurship engagement (Liñán and Chen**, **2009****)**	
CEE1	I am ready to do anything to re-start/ continue my business
CEE2	I will make every effort to re-start/ continue my business
CEE3	I am determined to create/continue a firm in the future
CEE4	I have very seriously thought of starting a firm
CEE5	I have the firm intention to start a firm someday
**New opportunity recognition (Kuckertz et al.**, **2017****)**	
NOR1	I am always alert to business opportunities (dropped)
NOR2	I research potential markets to identify business opportunities
NOR3	I search systematically for business opportunities
NOR4	I look for information about new ideas on products or services
NOR5	I regularly scan the environment for business opportunities

To increase the willingness and motivation of participants, this study provided rewards (such as mobile credit or e-wallet balance) to the first 20 participants who submitted valid questionnaires. Users were required to provide an e-mail address to ensure that they did not participate in the survey more than once. Data filtering was used to eliminate invalid responses. A total of 146 samples were collected in a valid final survey. Table 3 summarizes the descriptive statistics of the sample.

**Table 3:** Sample demographics.

**Characteristic**	**Items**	**Frequency**	**Percentage**
Gender	Male	66	45.2%
	Female	80	54.8%
	**Total**	**146**	**100%**
Age	16–20	15	10.3%
	21–25	43	29.5%
	26–30	39	26.7%
	31–35	21	14.4%
	>36	28	19.1%
	**Total**	**146**	**100%**
Education level	High school	35	24%
	Diploma/Bachelor	104	71.2%
	Master's	4	2.7%
	Doctoral	3	2.1%
	**Total**	**146**	**100%**

## Data Analysis

Two measures of evaluating and measuring partial least squares (PLS) were conducted. In the initial step, the validity and reliability analyses were carried out. Then, the coefficient path and the explanatory power of the structural model were tested. The goal of these two steps was to confirm the validity and reliability of the construct and to check the relationship between the constructs (Anderson and Gerbing, 1988; Hulland, 1999). PLS has been implemented and considered as the best tool for describing the causal interaction between construct variables and hence can concurrently handle model constructs and measurement items (Petter et al., 2007). In addition, since PLS has relatively simple parameters for variable normality and randomness, it is ideal for discussing relationships between variables in an irregular distribution of results. It can also evaluate dynamic prediction models (Chin and Newsted, 1999). Thus, PLS is more acceptable for this research than other SEM approaches to evaluate relationships between variables, eliminate measurement errors, and avoid collinearity.

### Outer Model and Scale Validation

The related external model measurements include the reliability and the internal consistency of each item, convergent validity, and discriminatory validity of each design. Appropriate question loading tested the reliability of the products. Factor loading was the expressive degree of determination, and a threshold value of 0.6 was used for individual reliability (Hair et al., 2016). Both observed variables follow the criteria after the elimination of any model. Table 4 indicates the composite reliability of each construct. For each construct, any composite-reliability (CR) rating higher than 0.7 (Chin, 1998) suggests that the construct was internally acceptable.

**Table 4:** Reliability analysis and convergent validity.

**Construct**	**Measurement item**	**Factor loading**	**Cronbach alpha**	**Composite reliability**	**AVE**
Entrepreneurial self-efficacy	ESE1	0.926	0.964	0.972	0.875
	ESE2	0.956			
	ESE3	0.946			
	ESE4	0.919			
	ESE5	0.929			
Internal locus of control	ILC1	0.921	0.956	0.966	0.85
	ILC2	0.935			
	ILC3	0.902			
	ILC4	0.929			
	ILC5	0.921			
Perceived learning from failure	PLF1	0.915	0.93	0.948	0.786
	PLF2	0.940			
	PLF3	0.930			
	PLF4	0.884			
	PLF5	0.750			
Recovery capability	REC1	0.929	0.917	0.942	0.801
	REC2	0.813			
	REC3	0.941			
	REC4	0.892			
	REC5	(deleted)			
Continuance entrepreneurship engagement	CEE1	0.874	0.903	0.929	0.723
	CEE2	0.890			
	CEE3	0.885			
	CEE4	0.869			
	CEE5	0.721			
New opportunity recognition	NOR1	(deleted)	0.948	0.963	0.866
	NOR2	0.941			
	NOR3	0.942			
	NOR4	0.950			
	NOR5	0.887			

Furthermore, the convergent validity test and the discriminant validity test were used to verify the construct validity. Fornell and Larcker (1981) proposed that the convergence validity could be verified when the factor loads of the metrics are >0.5, the average variance derived (AVE) is >0.5, and reliability is >0.7. Table 5 indicates that all constructs conform to the recommendations of Fornell and Larcker (1981), suggesting favorable convergent validity. Additionally, the discriminant validity was determined by comparing the square root of AVE to the correlation coefficient of the constructs. Tables 3, 4 show that the construct had discriminant validity.

**Table 5:** Correlation matrix.

	**CEE**	**ESE**	**ILC**	**NOR**	**PLF**	**REC**
CEE	0.850					
ESE	0.513	0.935				
ILC	0.568	0.874	0.922			
NOR	0.083	0.276	0.268	0.93		
PLF	0.647	0.837	0.903	0.305	0.887	
REC	0.635	0.656	0.746	0.212	0.778	0.895

*CEE, continuance entrepreneurship engagement; ESE, entrepreneurial self-efficacy; ILC, internal locus of control; NOR, new opportunity recognition; PLF, perceived learning from failure; REC, recovery capability*.

### Common Method Variance Testing

Common method variance (CMV) could be a major challenge for any self-reported data and SEM, which is used in this research data collection methodology. The existence of the CMV in the dataset means that the findings are not empirically correct. To control this issue, this research adopts Harman's one-factor test to test the existence of the CMV (Podsakoff and Organ, 2016). The exploratory factor analysis was conducted to verify that the first factor was <50% on all observed indicators. Explanatory variance for the first factor was 37.9%, indicating that CMV is not an issue in this research.

### Inner Model

The internal PLS model analysis was applied to analyze the hypotheses. The path coefficients are the direction and strength of the connection between the variables that imply cause and effect between the measured variables and the potential ones. Moreover, the *R* square value corresponds to the percentage of predictor variables that represent the predictive capacity of the model. Bootstrapping was used to estimate the degree of any path coefficient. The estimation was made by re-sampling data and the estimated values were more precise than the commonly used limit approximate value (Purvis et al., 2001). This study, therefore, used this approach to determine the significant relationship between variables.

**Figure 2:**
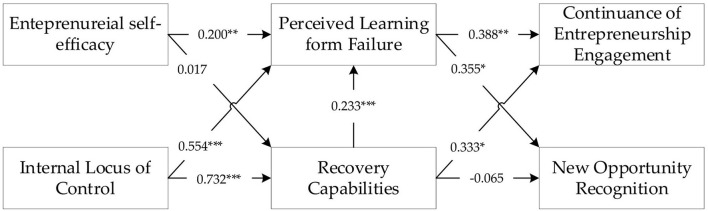
Framework of the inner model results. *p-value < 0.05; **p-value < 0.01; ***p-value < 0.001.

Table 6 shows that entrepreneurial self-efficacy has a positive effect on perceived learning from failure, supporting H1 (ESE → PLF: β = 0.200, *t*-value = 2.835); however, it has no significant effect on recovery capability (ESE → REC: β = 0.017, *t*-value = 0.128), means H2 not supported. Thus, the internal locus of control significantly impacts both perceived learning from failure and recovery capability, so H3 and H4 supported (ILC → PLF: β = 0.554, *t*-value = 7.435; ILC → REC: β = 0.732, *t*-value = 5.998). In contrast, recovery capability significantly affects perceived learning from failure, which supporting H5 (REC → PLF: β = 0.233, *t*-value = 5.585). H6 and H7 were supported because perceived learning from failure has a positive effect on continuance entrepreneurship engagement and new opportunity recognition (PLF → CEE: β = 0.388, *t*-value = 2.993; PLF → NOR: β = 0.355, *t*-value = 2.434). Finally, recovery capability has a significant impact on continuance entrepreneurship engagement, supporting H8 (REC → CEE: β = 0.333, *t*-value = 2.496), but does not have a significant impact on new opportunity recognition (REC → NOR: β = −0.065, *t*-value = 0.542), so H8 was not supported. Figure 2 illustrates the overall results of the inner model.

**Table 6:** Summary of the inner model result.

	**Hypothesis**	**Path coefficient**	***t*-value**	**Result**
H1	ESE → PLF	0.200^**^	2.838	Supported
H2	ESE → REC	0.017	0.128	Not supported
H3	ILC → PLF	0.554^***^	7.435	Supported
H4	ILC → REC	0.732^***^	5.998	Supported
H5	REC → PLF	0.233^***^	5.585	Supported
H6	PLF → CEE	0.388^**^	2.993	Supported
H7	PLF → NOR	0.355^*^	2.434	Supported
H8	REC → CEE	0.333^*^	2.496	Supported
H9	REC → NOR	−0.065	0.452	Not supported

*CEE, continuance entrepreneurship engagement; ESE, entrepreneurial self-efficacy; ILC, internal locus of control; NOR, new opportunity recognition; PLF, perceived learning from failure; REC, recovery capability*.

* *p < 0.05;*

** *p < 0.01; and*

*** *p < 0.001*.

*Number of bootstrap samples = 10,000*.

### Mediation Test

In this study, mediation tests were carried out to find out more about possible important effects of the proposed model. This study adopts the bootstrapping approach with bias-corrected confidence estimations to assess the effects of the mediators (Hayes and Preacher, 2014). Bootstrapping provides an empirical representation of the sampling distribution of the indirect impact by considering the generated sample of size *n* as a small representation of the population, which is continually resampled throughout the analysis to imitate the original sampling process (Hayes, 2009). Thus, bootstrapping is a valid and effective method for testing the mediation effect.

The results of mediation tests are presented in Table 7. There was a 95% CI of the specific mediating effects with 10,000 bootstrap resamples. The decision of the mediation effect is significant if the *t*-values and *p*-values are >1.96 and 0.05, respectively. Moreover, to obtain further results on the mediation analysis, a 95% bootstrapped CI bias is needed. The mediation effects exist if the indirect effect 95% bias-corrected bootstraps do not straddle a 0 in between (Preacher and Hayes, 2008).

**Table 7:** Mediation test.

**Relationship**	**Std. beta**	**Std. error**	***t*-value**	**Confidence interval** **(bias-corrected)**		**Decision**
				**LL**	**UL**	
ESE → PLF → CEE	0.078	0.036	2.135^*^	0.025	0.169	Supported
ESE → REC → CEE	0.006	0.048	0.118	−0.092	0.102	Not supported
ESE → PLF → NOR	0.071	0.042	1.672	0.008	0.178	Not supported
ESE → REC → NOR	−0.001	0.021	0.053	−0.056	0.036	Not supported
ILC → PLF → CEE	0.215	0.077	2.799^**^	0.086	0.388	Supported
ILC → REC → CEE	0.244	0.103	2.356^*^	0.056	0.469	Supported
ILC → PLF → NOR	0.197	0.086	2.297^*^	0.024	0.360	Supported
ILC → REC → NOR	−0.047	0.110	0.431	−0.270	0.168	Not supported

*CEE, continuance entrepreneurship engagement; ESE, entrepreneurial self-efficacy; ILC, internal locus of control; NOR, new opportunity recognition; PLF, perceived learning from failure; REC, recovery capability; LL, lower level; UL, upper level*.

* *p < 0.05; and*

** *p < 0.01*.

*Number of bootstrap samples = 10,000*.

The bootstrapping analysis shows that the relationship of entrepreneurial self-efficacy to continue entrepreneurship engagement through perceived learning from failure (ESE → PLF → CEE) was supported. However, other entrepreneurship self-efficacy relationships (ESE → REC → CEE; ESE → PLF → NOR; ESE → REC → NOR) are not supported. Furthermore, the relationships between the internal locus of control to continuance entrepreneurship engagement through perceived learning from failure and recovery capability (ILC → PLF → CEE; ILC → REC → CEE) are both supported. Moreover, the relationship of internal locus of control to new opportunity recognition through perceived learning from failure (ILC → PLF → NOR) is also supported. But the relationship of internal locus of control to new opportunity recognition through recovery capability (ILC → REC → NOR) is not supported.

## Discussion

This study considered how psychological characteristics underpin the desire of an individual to be an entrepreneur, can affect the ability of an entrepreneur to stay in business, and find new opportunities in the future. Internal locus of control and entrepreneurial self-efficacy were used as psychological traits and combined with perceived failure learning and recovery capability as the post-failure behavior of entrepreneurs. Furthermore, as a result of entrepreneurship failure, continued entrepreneurship engagement, and new opportunity recognition were adopted. The empirical findings and contributions of this study have significant implications for both academics and practitioners.

### Theoretical Implications

This study adds to the knowledge system about entrepreneurship resilience in several ways. First, we presented a comprehensive conceptual model for the outcomes, which include continued entrepreneurship engagement and recognition of new opportunities following entrepreneurship failure. Despite increased research interest in entrepreneurship, there has yet to be developed a holistic model that qualitatively explains entrepreneur failure and resilience. Second, this is the first study to link psychological characteristics of entrepreneurship intention to entrepreneur behavior after failure, which leads to failure outcomes. By utilizing the new data form, this study makes several contributions to the related field of research.

### Managerial Implications

The findings shed light on how entrepreneurs manage their psychological characteristics by concentrating on what they should do in the event of a failure. The findings suggest that entrepreneur stakeholders, such as investors, suppliers, employees, and customers, should pay attention to the psychological traits that underpin entrepreneurship, particularly internal locus of control and entrepreneurial self-efficacy. These psychological traits can be used by stakeholders to assess the ability of an entrepreneur to respond to a failure to anticipate stakeholder losses. Thus, the findings of this study show that positive emotions are also important in coping with and recovering from failure.

The hypothesis 1 test found that entrepreneurial self-efficacy significantly impacts perceived learning from failure. Then, the internal locus of control has a positive influence on perceived learning from failure and recovery capability, so hypotheses 3 and 4 are supported. This finding suggests that entrepreneurs keep their positive self-efficacy and internal locus of control because these psychological traits indicate their ability to learn from failure and recover after failure. The stakeholders of an entrepreneur can indicate the confidence of an entrepreneur and their belief that every effort by them will support their ability to learn from failure in support of recovery. However, the result indicates that entrepreneurial self-efficacy has no significant impact on recovery capability. This is because once failure occurs, confidence will decrease, and individuals need time to recover. Omorede (2020) found that positive emotions can either expedite or hinder recovery from failure. In other words, entrepreneurs may still be recovering from a failure that has occurred so that it is difficult to recover.

The test results of hypothesis 5 show that recovery capability significantly impacts perceived learning from failure. Amankwah-Amoah et al. (2016) discovered that the start of their learning process was the phase of grief for entrepreneurs who have failed. This result indicates that entrepreneurs will learn effectively from their failure experience after they have fully recovered. The stakeholders of an entrepreneur can evaluate the recovery process of an entrepreneur, and the faster the entrepreneur recovers, the sooner they will learn from their failures and will open up other opportunities.

According to the findings, entrepreneurs generally either look for potential business opportunities or invest in a related business after failure. This is in line with the testing result of hypotheses 6, 7, and 8 that state perceived learning from failure supports returning to entrepreneurship and seeking new opportunities, and that recovery capability supports continued entrepreneurship. This result suggests to the stakeholder that the ability of an entrepreneur to learn from failure and their recovery capability can be an indication of their commitment to entrepreneurship, either creating a new business or restarting the failed one. Moreover, their ability to learn from failure also enhances the chance to find a new opportunity that can support a future business. Entrepreneurs need to increase their ability to learn from failure because even the equity funds want to invest in companies that have failed before (Cope, 2011). However, the testing results of hypothesis 9 shows that recovery ability has a non-significant impact on new opportunity recognition. This study argues that the recovery process may distract the thinking process of entrepreneurs, which restricts their ability to find new opportunities.

## Conclusion and Future Study

This study proposes a model to explain how entrepreneurs react to failure based on their psychological characteristics and why the psychological characteristics that underlie the intention of an individual to be an entrepreneur remain as the basis for them to recognize new opportunities and continue their engagement and entrepreneurship. In general, results show that psychological characteristics represented by an internal locus of control and entrepreneurial self-efficacy can support the willingness of entrepreneurs to gain from failure and raise their recovery capabilities, increasing their willingness to continue entrepreneurship and helping them to recognize new opportunities. If entrepreneurs can better understand the failure experience and process, they may derive more insight into methods they can adopt in future ventures and new opportunities. However, recovery capability will not be influenced by entrepreneurial self-efficacy and will not influence new opportunity recognition. This may be because positive emotions do not necessarily speed up the recovery process from failure (Omorede, 2020), so recovery ability may vary among entrepreneurs depending on many factors.

This study enriches the literature on entrepreneurship failure, which is currently dominated by qualitative studies, rather than the quantitative approach in this study. Furthermore, this research guides stakeholders related to entrepreneurship on how to conduct an early assessment of their partners, and as a result, they can minimize the risk of a future failure.

Despite the efforts to develop a comprehensive conceptual model and analyze new, various flaws could guide future studies. First, this research only focuses on the internal locus of control and entrepreneurial self-efficacy, although there are many other psychological traits that influence the intention of an entrepreneur. Future research could consider other psychological traits for more comprehensive results. The psychological traits in this research describe only the internal factors of the entrepreneur, and future research could explore external factors that influence entrepreneurial intention. Third, although the methods employed in this research are adequate, other methods also need to be explored to find more impactful conclusions. Fourth, future research could adopt other research methods to explain reciprocal or opposite direction relationships between the variable and deepen the findings both theoretically and managerially. Finally, future studies should look at how positive emotions can speed up or slow down the recovery process from failure, as well as under what conditions positive emotions lead to positive results after a failure experience.

## Data Availability Statement

The original contributions presented in the study are included in the article/Supplementary Material, further inquiries can be directed to the corresponding author/s.

## Author Contributions

Conceptualization, methodology, writing—original draft preparation, and validation by HZ. Formal analysis, investigation, visualization, writing—review, and editing by AW. Both authors have read and agreed to the published version of the manuscript.

## Funding

This study appreciated the partial financial support from the Planning Office of Philosophy and Social Sciences of Heilongjiang Province, China (Grant Number: 19ZWD214).

## Conflict of Interest

The authors declare that the research was conducted in the absence of any commercial or financial relationships that could be construed as a potential conflict of interest.

## Publisher's Note

All claims expressed in this article are solely those of the authors and do not necessarily represent those of their affiliated organizations, or those of the publisher, the editors and the reviewers. Any product that may be evaluated in this article, or claim that may be made by its manufacturer, is not guaranteed or endorsed by the publisher.
